# Redox‐Responsive Hydrogels Loaded with an Antibacterial Peptide as Controlled Drug Delivery for Healing Infectious Wounds

**DOI:** 10.1002/adhm.202401289

**Published:** 2024-07-08

**Authors:** Mariam Cherri, Paraskevi S. Stergiou, Zainab Ahmadian, Tatyana L. Povolotsky, Boonya Thongrom, Xin Fan, Ehsan Mohammadifar, Rainer Haag

**Affiliations:** ^1^ Institute of Chemistry and Biochemistry Freie Universität Berlin Takustr. 3 14195 Berlin Germany; ^2^ Department of Pharmaceutics School of Pharmacy Lorestan University of Medical Sciences 68151-44311 Khorramabad Iran

**Keywords:** antimicrobial drug delivery, biomaterials, hyperbranched polyglycerol, reducible hydrogels

## Abstract

Infectious wounds occur when harmful microorganisms such as bacteria or viruses invade a wound site. Its problems associated include delayed healing, increased pain, swelling, and the potential for systemic infections. Therefore, developing new wound dressing materials with antibacterial effects is crucial for improving the healing process. Here a redox‐degradable hydrogel loaded with an antibacterial peptide (vancomycin) in a straightforward gram‐scale synthesis, is developed. The hydrogel structure consists of a disulfide bond‐containing hyperbranched polyglycerol (SS‐hPG) that is cross‐linked by 4‐arm polyethylene glycol‐thiol (4‐arm PEG‐SH). The polymerization mechanism and full characterization of SS‐hPG are described as this synthesis is reported for the first time. Rheology is used to ascertain the hydrogel's mechanical characteristics, such as stiffness, and self‐healing, determining these properties for different ratios and concentrations of both gel components. The incorporation of disulfide bonds in the hydrogel is proved by conducting degradation experiments in reductive environments. Fluorescein isothiocyanate‐albumin (FITC‐BSA) and vancomycin both are loaded into the gel, and the guest release kinetics is assessed for both slow and on‐demand releases. Finally, the in vitro and in vivo experiments prove that the vancomycin‐loaded hydrogel acts as an antibacterial barrier for wound dressing and accelerates the healing of infectious wounds in a mouse model.

## Graphical abstract

A redox‐degradable hydrogel loaded with vancomycin is developed using a straightforward gram‐scale synthesis. The hydrogel, composed of disulfide bond‐containing hyperbranched polyglycerol (SS‐hPG) and 4‐arm polyethylene glycol‐thiol (4‐arm PEG‐SH), demonstrates slow and on‐demand release kinetics. In vitro and in vivo experiments confirm its effectiveness as an antibacterial barrier and its ability to accelerate wound healing in a mouse model.

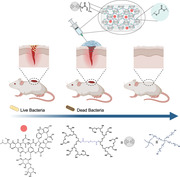

## 1 Introduction

The most important function of the skin, as the first line of defense, is to protect the body against microbial invasions. However, as the outermost organ, it is always vulnerable to damage caused by wounds. Injured skin offers a suitable site for the accumulation and proliferation of bacteria, leading to infection and delaying wound healing.^[^ ^1^ ^]^ The optimal process of wound healing can be divided into four phases, which occur interdependently and simultaneously: (i) vasoconstriction and coagulation, collectively leading to hemostasis, (ii) acute inflammation, (iii) cellular proliferation, and (iv) wound remodeling.^[^ ^2^ ^]^ In proliferation, new extracellular matrix (ECM), collagen and fibronectin by fibroblasts, their differentiated counterparts, and the myofibroblasts are deposited.^[^ ^2^, ^3^ ^]^ Acute inflammatory response is a crucial stage in the course of wound healing, providing early protection against bacteria by first recruiting pathogen‐destroying phagocytic neutrophils and later recruiting macrophages.^[^ ^2^, ^3^ ^]^ Since long‐term inflammatory responses are correlated to chronic wounds, bacterial infection is one of the most serious complications affecting wound healing and tissue regeneration. The treatment of these infections usually consists of the continuous administration of oral or intravenous antibiotics over a period of weeks or months. This can lead to inefficient antibiotic delivery to the target site, which in turn can cause, in severe cases, sepsis – an infection throughout the entire body.^[^ ^4^ ^]^ Furthermore, applying antibiotics to the body as a whole, and not only the target area, also causes collateral damage by killing the natural microbiota of the host. This effect can lead to further weakening of the immune system, allowing pathogenic or antibiotic‐resistant bacteria to dominate the colonization of the host.^[^ ^5^ ^]^ These challenges highlight the need for topical delivery systems that can increase antibiotic effectiveness by releasing it locally to the targeted wound site while reducing side effects by decreasing the dosage.

To accelerate the healing process, especially for extensively damaged chronic wounds of the types caused by injury or specific diseases, wounds should be covered by an effective dressing material, with features such as high biocompatibility, ability to retain moisture, and mechanical properties well‐suited to injured tissue and antibacterial action.^[^ ^6^ ^]^ Conventional wound dressings, such as gauze, must be changed frequently, and the risks of infection, secondary damage during removal, and their lack of biological properties for wound healing hamper their usage.^[^ ^7^ ^]^ Hydrogels have gained extensive interest for wound dressing and healing applications due to several outstanding properties, such as wound adhesion, moisture retention, similar stiffness to skin, resemblance to biological tissues, and biocompatibility, all of which provide favorable conditions for wound healing. Additionally, the three‐dimensional (3D) porous structure of hydrogels provides a suitable scaffold for loading antibacterial drugs, which can then be released and delivered in a controlled process. The drug release is achieved through either diffusion or the passive degradation of hydrogel.^[^ ^8^ ^]^ Eventually, such degradation enables the dressing material to be removed from the wound without secondary damage.

Degradable hydrogels are typically prepared by introducing a stimuli‐responsive moiety into the backbone of a gel macromonomer, allowing degradation in response to stimuli such as pH, temperature, enzyme, redox state, etc.^[^ ^9^ ^]^ This stimuli‐responsive hydrogel degradation serves as a stimulus to release therapeutic agents in a controlled manner, or to ensure the hydrogel's ultimate removal in cases when it is used as a bandage for infected wounds. Among different approaches for “on‐demand” degradable hydrogels, the redox‐responsive approach has directed much interest toward polymer‐based hydrogels, which are typically synthesized by introducing disulfide bonds in a polymer backbone. Disulfide bonds can be cleaved in response to variations in environmental redox state.

In particular, redox‐responsive hydrogels show enhanced degradation in response to the overexpression of glutathione (GSH) or reactive oxygen species (ROS) produced in the wound, inflammation, or bacterial infection sites and biofilms.^[^ ^10^ ^]^ The redox imbalance that prevails in the infected wound environment can stimulate therapeutic agent release and hydrogel degradation. Recently, multiple redox‐responsive hydrogels as disulfide bond reservoirs have been reported for improving wound healing, with applications including the topical delivery of therapeutic agents, mainly proteins.^[^ ^11^ ^]^ Incorporation of disulfide bonds in the hydrogel structure is beneficial in several different aspects; (i) eventual degradation and removal of the hydrogel without causing secondary damage to the wound, (ii) providing dynamic bonds for self‐healing and (iii) adjusting the ROS level in the wound for mediation of redox potential to achieve higher wound healing efficacy.^[^ ^12^ ^]^

**Figure 1:**
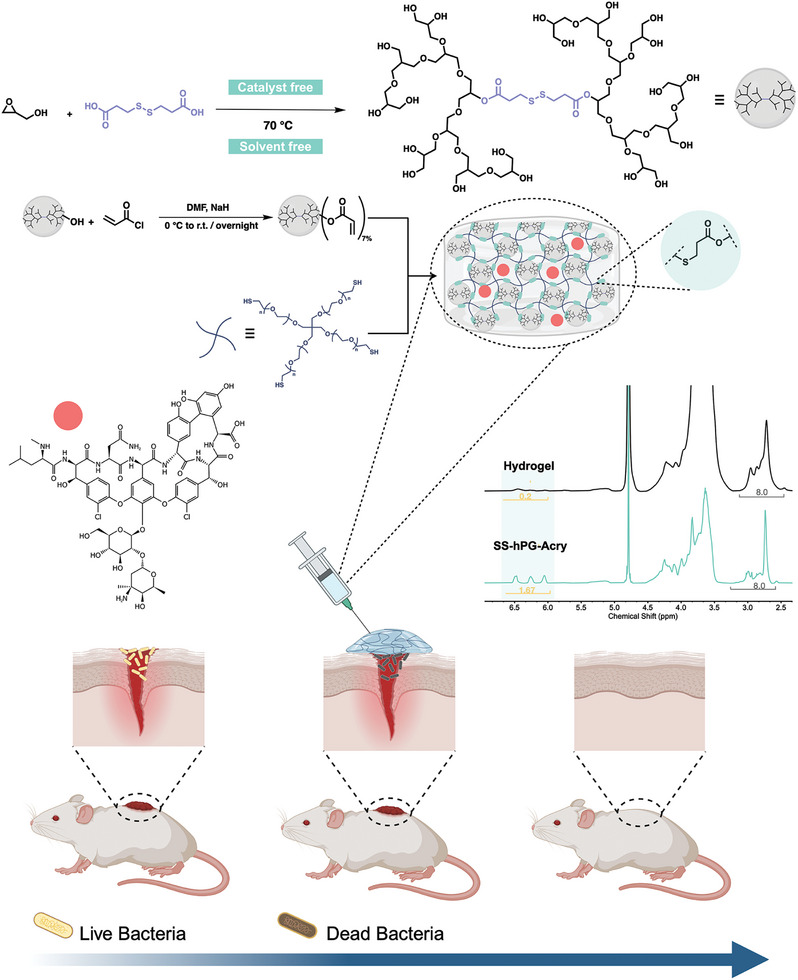
Overall scheme representing the synthesis of the hPG building block in addition to its acrylation and the hydrogel formulation. ^1^H NMR verifies the formation of the hydrogel through the disappearance of the acrylate correspondent peaks, confirming the thiol‐ene click reaction. The hydrogels were then loaded with vancomycin and applied over wounds in mice, destroying the existing bacteria and facilitating wound healing.

In this work, we present a one‐step straightforward synthesis of a disulfide bond‐containing hyperbranched polyglycerol macromonomer (SS‐hPG). The synthesis is performed in a catalyst‐ and solvent‐free process on a 30 g scale. The synthesized SS‐hPG is employed as a building block of a redox‐responsive hydrogel that is chemically crosslinked via 4‐arm‐PEG‐SH through thiol‐Michael addition click reaction. An antibacterial peptide, vancomycin, was loaded in the redox‐responsive hydrogel to be released either gradually or on demand. Crucially, the viscoelastic properties, mechanical strength, degradation of SS‐hPG, and the release time of the loaded antibiotic, can be tuned by varying the concentration of the polymeric components in the hydrogel (**Figure** **1**).

## 2 Results and Discussion

### 2.1 Synthesis of Redox‐Responsive Hyperbranched Polyglycerol

As one of the hydrogel components, the redox‐responsive hyperbranched polyglycerol was achieved on a 30 g scale through the cationic polymerization of glycidol using 3,3′‐dithiodipropanoic acid (DTDPA) as a monomer activator in a solvent‐free reaction under mild conditions. (Figure S1A, Supporting Information). DTDPA, with its two carboxylic acids (pK_a_ 3.9), can activate the epoxide ring of glycidol toward cationic polymerization. Since DTDPA has two carboxylic acid groups on each end, the polyglycerol backbone could propagate telechelically, ending up with one molecule of DTDPA in the center of the hyperbranched polyglycerol backbone. The suggested mechanism was an activated‐monomer (AM) cationic ring opening polymerization (Scheme S1, Supporting Information), starting with proton transfer from the DTDPA's carboxylic acid groups to the epoxide ring, resulting in an activated glycidol. The deprotonated carboxylates of DTDPA could further open the activated epoxide rings, resulting in its incorporation in the polymer backbone. The reaction mechanism of carboxylic acid‐containing molecules and glycidol has been fully investigated in our group's previously reported work, in which we copolymerized citric acid and glycidol.^[^ ^13^ ^]^ We should mention that the hydroxyl group of glycidol could also initiate the ring opening, as hydroxyl groups are more reactive in nucleophilic attack than carboxylate,^[^ ^14^ ^]^ but due to the high concentration of DTDPA and the high reactivity of protonated glycidol, carboxylate groups can also initiate the polymerization.

To study the effect of DTDPA on the polymer structure, experiments with different molar ratios of glycidol to DTDPA [Gly]:[DTDPA]; 5, 10, and 20 were carried out at 70 °C. The respective products of these experiments are called hPG_5_‐DTDPA, hPG_10_‐DTDPA, and hPG_20_‐DTDPA. The polymers were fully characterized using various spectroscopic and analytical methods to verify the incorporation of DTDPA in the polymeric backbone, quantify the disulfide content, and identify the degree of branching of the hyperbranched polyglycerol. The proton signals at 2.6‐3.0 ppm are assigned to the methylene groups adjacent to the disulfide and ester‐carbonyl in ^1^H NMR and can be used as the first proof of the integration of DTDPA in the polymer structure. The decrease in the intensity of the ester‐carbonyl stretches when decreasing the amount of initiator in the feed enforces the participation of DTDPA in the polymerization (Figure S1B, Supporting Information). In FTIR, the absorbance of the ester‐carbonyl stretch was present at 1729.6 cm^−1^ (Figure S1C, Supporting Information). By comparison, the value for the carboxylic acid‐carbonyl stretch of DTDPA was 1630.2 cm^−1^ (Figure S2, Supporting Information). Figure S1D (Supporting Information) also shows the inverse‐gated ^13^C NMR spectrum of the resulting polymer. We identified the different structural units and calculated their relative abundance as well as the degree of branching of AB_2_ type monomers (Table S1, Supporting Information), as reported by Hölter and Frey.^[^ ^15^ ^]^

The molecular weight of the resulting polymers is also reported in Table S1 (Supporting Information), as calculated from ^1^H NMR (Equation S1, Supporting Information). For chain growth polymerizations, including cationic polymerization, the degree of polymerization can be related to the kinetic chain length, which is the result of the number of consumed monomers per initiated chain.^[^ ^16^ ^]^ Therefore, increasing the initiator concentration will lead to a decrease in the degree of polymerization, and hence a decrease in the average molecular weight of the polymer. hPG_5_‐DTDPA, with its 5:1 ratio of monomer to the initiator, has a lower molecular weight than hPG_20_‐DTDPA with its 20:1 ratio. On the other hand, the degree of branching is inversely proportional to the ratio of DTDPA present in the feed. This may be related to the availability of many glycidol monomers that can be activated in the presence of a larger amount of DTDPA – and therefore the increased possibility of reaction between the secondary hydroxyl groups and other monomers.

We observed from the inverse gated ^13^C NMR spectrum (Figure S1D, Supporting Information) that the relative abundance of the L_1,4_ structural units was high compared to the obtained L_1,4_ structural units when glycidol was polymerized in an anionic fashion. The excessive presence of L_1,4_ structural units also indicates that the mechanism of polymerization was through the activated monomer (AM) mechanism, where the glycidol monomers were first activated by carboxylic acid groups before undergoing the ring opening polymerization. We also demonstrated that DTDPA was incorporated in the dendritic polyglycerol core through two‐dimensional (2D) heteronuclear multiple bond connectivity (HMBC) NMR (Figure S1E, Supporting Information). The correlation between the proton signals of the polyglycerol backbone at 3.5 and 4.2 ppm and the ester‐carbonyl signal at 180 ppm was an indication for the existence of DTDPA in the polymeric core. To prove that the DTDPA has a major role in the polymerization of glycidol, we performed a control reaction under the same reaction conditions (catalyst free, solvent free, and at 70 °C), but without DTDPA. After performing dialysis with a membrane of a molecular weight cutoff (MWCO) of 1 kDa, the reaction yield was negligible (less than 5%). Hence, we could conclude that DTDPA was essential in initiating the reaction.

We aimed to prove that the hyperbranched polyglycerol with DTDPA can undergo degradation under reductive conditions before incorporating it as a building block in a hydrogel. We wanted to present a proof of concept that the disulfide bond can be reduced in the presence of a reducing agent while also identifying the resulting degradation products. hPG_10_‐DTDPA, referred to as SS‐hPG throughout the remainder of this manuscript, was incubated in an aqueous solution containing 10 mM of 3,3′,3′′‐Phosphanetriyltripropanoic acid (TCEP) as reducing agent to cleave the disulfide bonds. After 3 days, ^1^H NMR was used to identify the resulting degradation products (Figure S3, Supporting Information). In the ^1^H NMR of degradation products, the peak at 3.0 ppm, assigned to the methylene protons adjacent to the disulfide bond, disappeared, while a new peak appeared at 2.9 ppm, which is assigned to the methylene protons adjacent to thiol groups. Hence, our degradation products are a polyglycerol chain with a free thiol group, a polyglycerol chain completely detached from DTDPA due to the ester cleavage, and 3‐mercaptopropanoic acid resulting from the cleavage of both ester and disulfide groups.

### 2.2 Formation and Characterization of the Hydrogel

To achieve a cross‐linkable SS‐hPG, 7% of the hydroxyl groups were converted into acrylate groups by esterification with acryloyl chloride to obtain SS‐hPG‐Acry. The hydrogel was subsequently formed by mixing the PBS solution of SS‐hPG‐Acry and 4‐arm‐PEG‐SH with a determined concentration after overnight incubation at room temperature. The hydrogels were prepared at different weight percent concentrations to investigate the effect of gel concentration on the stiffness and mechanical properties of the hydrogels. The hydrogels were prepared by chemical cross‐linking of the thiol groups of the 4‐arm‐PEG‐SH and the acrylates of the SS‐hPG‐Acry via a thiol‐Michael addition click reaction (Figure S7A, Supporting Information). After gelation, the ^1^H NMR spectrum showed 90% reduction in the peaks associated with the protons of the alkene group of the acrylate in the region between 6.0 and 6.6 ppm (Figure S7B, Supporting Information) after 1 h. When observing the corresponding FTIR spectra of the compounds, an alkene band appeared after acrylation at 800 cm^−1^ and disappeared in result of gelation. The thiol stretching absorbance band at 2700 cm^−1^ disappeared as well in the hydrogel spectra, indicating the thiol‐ene click reaction.

**Figure 2:**
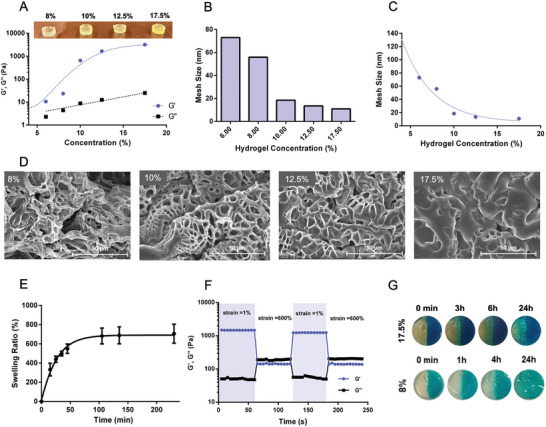
A) Elastic and viscous modulus values (G′ and G″) of different concentrations of reducible hPG block and 4‐arm‐PEG‐SH crosslinker for a 1:1 ratio. B) G′ and G″of hydrogels for different reducible hPG block and 4‐arm‐PEG‐SH crosslinker ratios at a concentration of 17.5%. C) Mesh size of hydrogels with different components’ concentrations at 1:1 ratio according to the Canal‐Peppas equation. D) SEM images of freeze‐dried hydrogels with different concentrations of the building components. E) Swelling ratio of 17.5% hydrogel. F) G′ and G″of 17.5% hydrogel measured in cycles of 1% and 600% strain. G) Photos taken at different time points of 17.5% and 8% hydrogel, one half without dye and second half with blue dye, combined together to prove the self‐healing properties of the hydrogel.

**Figure 3:**
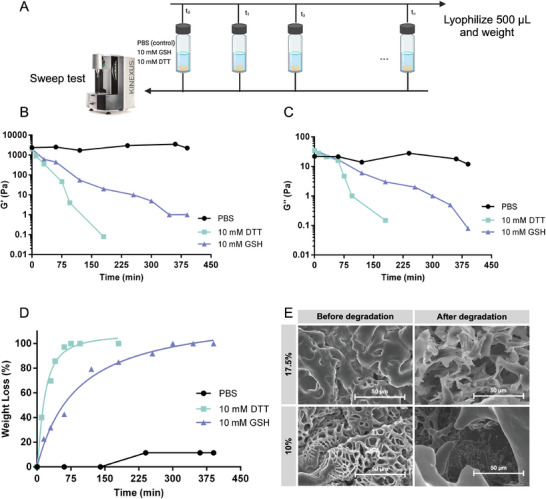
A) Scheme representing the degradation experiment. Several 17.5% hydrogels were incubated in 1 mL of media (PBS, 10 mM GSH, and 10 mM DTT). At each time point, G′ and G″ were measured, and 500 µL of the solution media was lyophilized and weighed to identify the loss in the hydrogel weight. B–D) Degradation of the 17.5% hydrogels identified by recording G′, G″ and the hydrogel weight loss respectively, at different time points, in GSH or DTT media. E) SEM images of freeze‐dried 10% and 17.5% hydrogels recorded before and after incubation with 10 mM DTT for 24 h.

Redox‐responsive hydrogels were prepared by mixing SS‐hPG‐Acry and 4‐arm‐PEG‐SH in a 1:1 molar ratio at varying weight concentrations (%w/v) of (6%, 8%, 10%, 12.5%, and 17.5%). The rheological properties of the hydrogels were compared through the elastic and viscous modulus values G′ and G″, respectively. G′ and G″ provide insights into how materials respond to deformation under oscillatory shear. G' indicates the solid‐like behavior (elasticity), while G″ indicates the liquid‐like behavior (viscosity). G′ and G″ are calculated from the frequency sweep test, where the shear strain is fixed at 1% and the G′ and G″ values are set according to the linear region when varying the frequency. The gel with lower concentrations of polymeric content exhibited lower G′ and G″and behaved as a soft gel. At increased concentrations a noticeable increase in G′ and G″was observed, along with increased gel stiffness (**Figure** **2**A). The mesh size of the hydrogels was calculated according to the Canal‐Peppas equation, with G′ as the varying parameter. Figure 2C, showing the results of the calculations, expresses a trend of decreasing mesh size with increased polymeric content concentration; in other words, the mesh size was inversely proportional to G′. Recorded SEM images of freeze‐dried hydrogels showed smaller hydrogel mesh size for hydrogels with higher concentrations of SS‐hPG‐Acry and 4‐arm‐PEG‐SH (**Figure** **3**D). The elastic and viscous moduli were also tested when fixing the concentration at 17.5% and varying the SS‐hPG‐Acry:4‐arm‐PEG‐SH ratio. Gels did not form for a 3:1 ratio. The values of G′ and G″ increased drastically for a ratio of 1:1 and almost plateaued when further increasing the 4‐arm‐PEG‐SH to SS‐hPG ratio (Figure 2B). Moreover, the 17.5% gel was able to swell from its dried state, showing an almost eightfold weight increase over two hours (Figure 2D). The gels exhibited self‐healing properties due to the hydrogen bonding, as well as the dynamic nature of the disulfide bonds. To investigate these self‐healing properties, extreme strain (600%) and mild strain (1%) were applied to the 17.5% hydrogel in alternating cycles (Figure 2E). Under 600% strain the gel was totally deformed: G′ dropped approximately tenfold and became even lower than G″. G′, as a representative of the crosslinked network density, gets lower because the network collapses, losing the storage energy which is normally stored via the crosslinked network while G″ gets higher due to the applied energy that dissipates due to the lower network density which collapses when applying high shear strain force. However, when strain was reduced back to 1%, the gel showed instant recovery, with G′ and G″ returning to their original values. Thus, the hydrogels were able to conserve their viscoelastic properties and mechanical strength after their rupture if they remained partly intact. The self‐healing property was also be tested visually by staining one hydrogel with a blue dye and leaving the other one uncolored. Both hydrogels were cut in half and brought in contact, then observed at different time points (Figure 2F). The test was performed on both 8% and 17.5% hydrogels, confirming that the gels were able to merge again, and that the 8% gel was able to heal faster. The rheological properties of hydrogel after self‐healing were similar and comparable with the original hydrogel (Figure S8, Supporting Information). To prove the contribution of disulfide bonds in the self‐healing process the hydrogels were prepared in 2.5 mM urea solution in PBS that is known to hinder the H‐bonding. As shown in the photographs in Figure S9 (Supporting Information), the hydrogels prepared in urea solution did not self‐heal as efficiently as the ones prepared in PBS. This indicates that both H‐bonding and disulfide bonds are essential for self‐healing process.

### 2.3 Degradation and Release Properties of the Hydrogel

Degradation experiments have been performed to investigate the successful incorporation of disulfide bonds in the hydrogel structure. For this aim hydrogels were incubated in a reducing environment (10 mM GSH or 10 mM dithiothreitol, DTT) and PBS as control. At different time intervals, determined aliquots of the media were withdrawn and lyophilized to calculate the weight loss of the hydrogel upon degradation, and the storage and loss moduli of the hydrogel were measured by applying the time sweep test (Figure 3A). The hydrogel was completely deformed when incubated in a reducing environment: the values of G′ and G″ decreased tremendously. The degradation in DTT was twice as fast as the degradation in GSH, and the hydrogel was able to conserve its mechanical properties in the control medium (Figure 3B,C). The degradation was also proven by measuring the weight loss of the hydrogel over time. When the disulfide bonds were reduced, the hydrogel gradually lost the crosslinked network configuration, which allowed for the degraded segments to become soluble in the reducing media. Once the solution was lyophilized, the weight of those segments was calculated and the degradation profile of the hydrogels versus time was plotted (Figure 3D). The SEM images of freeze‐dried hydrogels (10% and 17.5% concentrations) recorded before and after incubation with 10 mM DTT show a deformation in the hydrogel network where the pore's configuration was disrupted; hence, we were able to visualize the consequential degradation of the hydrogel's network (Figure 3E).

**Figure 4:**
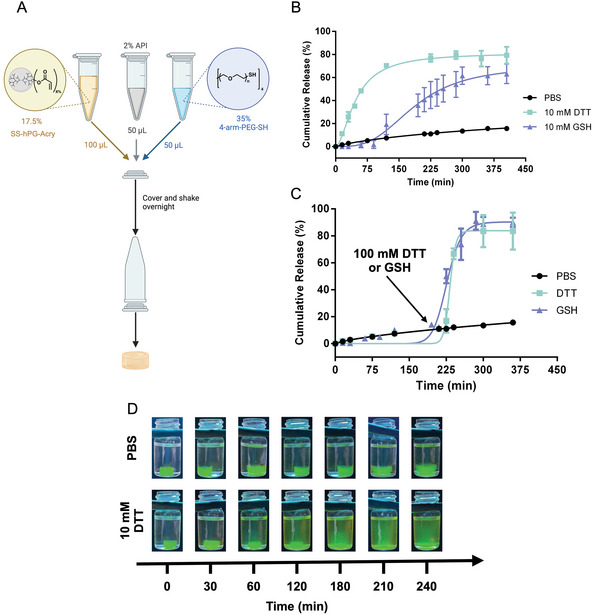
A) Scheme representing the encapsulation of active pharmaceutical ingredient (FITC‐BSA or vancomycin) in the hydrogel. B,C) slow and on‐demand release profiles, respectively, of FITC‐BSA‐loaded 17.5% hydrogel incubated in PBS, 10 mM GSH and DTT for on‐demand release. The hydrogel was incubated in PBS and a high concentration of GSH or DTT was added after 3 h. D) photos of FITC‐BSA‐loaded 17.5% hydrogel incubated in PBS and 10 mM DTT, captured at different time points.

Following the degradation of the hydrogels in a reductive environment, a model protein, albumin−fluorescein isothiocyanate conjugate (FITC‐BSA), was encapsulated in the hydrogel's network to evaluate the triggered protein release, either spontaneously or on‐demand, by adding high concentrations of the reducing agent at a certain time point. The encapsulation of FITC‐BSA was performed during the formation of the hydrogel by adding a solution of 2% wt. FITC‐BSA during the mixing of the hydrogel's components, as shown in **Figure** **4**A. As for the release profiles, the hydrogel could successfully release the FITC‐BSA in a reductive environment with the presence of either 10 mM GSH or DTT in a controlled fashion. The hydrogel barely leached any FTIC‐BSA in PBS (<20%), which indicates that the release was due to degradation of the hydrogel backbone, not physical diffusion (Figure 4B). In another experiment, the hydrogels were only incubated in PBS solution, and after 3 h, a high concentration of GSH or DTT was added to the media to observe the sudden release of FTIC‐BSA from the hydrogels. This proved that the release from the hydrogel can also occur on demand (Figure 4C). In Figure 4D, we took photographs under UV illumination (λ = 665 nm) of FTIC‐BSA‐loaded hydrogels in PBS and 10 mM DTT solutions over the course of 4 h. The hydrogel incubated in PBS remained intact. However, the hydrogel in the reducing environment completely dissolved and the entire media became fluorescent, indicating the release of the FITC‐BSA from the hydrogel after complete network degradation. Considering the elevated concentrations of ROS in the wound site together with the potential cleavage of disulfide bonds in oxidative environments,^[^ ^10^, ^17^ ^]^ the degradation of FTIC‐BSA‐loaded hydrogels in 10 mM H_2_O_2_ solution was conducted. As it can be seen in Figure S10 (Supporting Information), the hydrogels were degraded over 7 days which has much slower degradation rate compared to that in the reductive environment.

### 2.4 Cell Viability and In Vitro Antibacterial Activity

**Figure 5:**
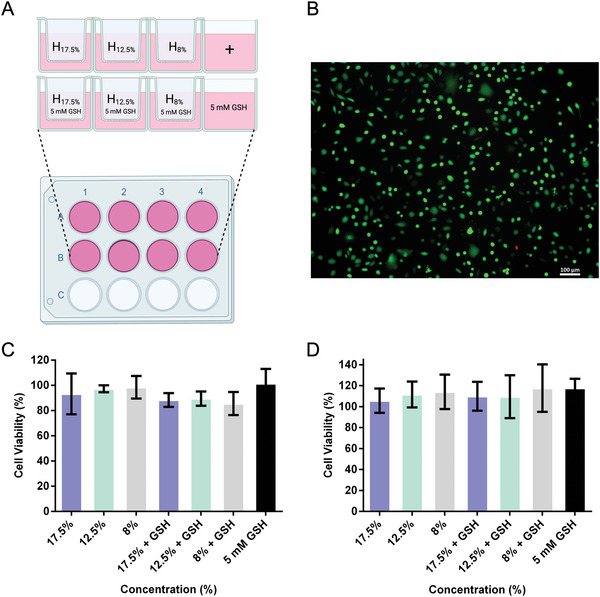
A) Scheme of the cytotoxicity assay setup, where a Transwell™ insert containing different hydrogel concentrations was inserted on top of the plate's well, with and without the addition of 5 mM GSH. B) Fluorescence microscopy images of the live/dead cell viability assay upon treatment with the 17.5% hydrogel after 24 h. C,D) Cell viability assays of cells treated with different hydrogels, with and without the addition of GSH, after 24 h and 96 h respectively.

The biocompatibility of the hydrogels with different concentrations (17.5%, 12.5%, and 8%) was tested on L929 mouse fibroblast cells using PrestoBlue cell viability assay. The experiment was set up to mimic a real‐life skin model by inserting a Transwell cell culture insert on top of the cell media to act as a porous barrier between the cells and the hydrogel (**Figure** **5**A). In addition, a solution of 5 mM GSH was added to the hydrogels to trigger degradation and to allow investigation of the degradation products’ cytotoxicity. Figure 5B shows a live/dead assay result in the form of a microscopic fluorescent image recorded for the cells incubated with 17.5% hydrogel after 24 h. Incubated cells were stained with a mixture of calcein‐AM and propidium iodide dyes, which differentiated their live or dead state through green and red fluorescence, respectively. The remarkably dominant green color showed that a great majority of the cells remained alive. As for the PrestoBlue assay tested on the variable concentrations and degradation products of the hydrogels, cells showed excellent viability after 24 h as well as 96 h, which demonstrated the high biocompatibility of the systems.

**Figure 6:**
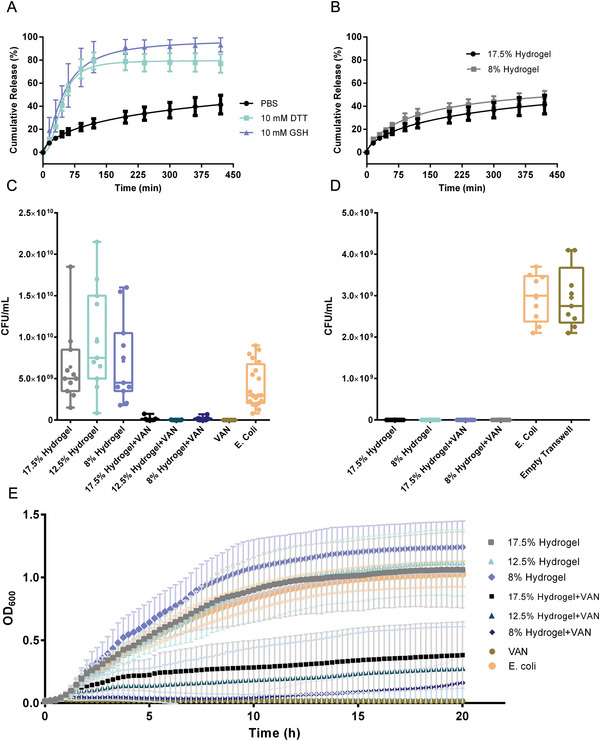
A,B) Release profiles of vancomycin‐loaded hydrogels in different media for the same 17.5% concentration and in PBS for the 17.5% and 8% hydrogels in PBS only. C) Antibacterial assays measurement of CFU after 20 h incubation with bacteria. D) Bacterial blockage. E) Bacterial growth curve showing long‐lasting antibacterial activity.

We envision these hydrogels serving as a delivery platform to ensure the controlled topical transport of active pharmaceutical ingredients. We therefore encapsulated vancomycin in the hydrogel network during hydrogel preparation. Vancomycin is a glycopeptide antibiotic medication used to treat bacterial infections. It is applied intravenously as a treatment for complicated skin infections, bloodstream infections, endocarditis, and bone and joint infections. To treat infected skin tissues, vancomycin is administrated intravenously in high doses to achieve effective results.^[^ ^18^ ^]^ The high doses often lead to challenges such as kidney failure, local pain, thrombophlebitis, and even anaphylaxis or erythema; all the while potentially contributing to resistance development.^[^ ^19^ ^]^ Therefore, delivering vancomycin topically can be an alternative option to overcome those challenges. The release profiles of vancomycin‐loaded hydrogels were plotted for the different reductive media and the control medium and were observed to show a controlled trend (**Figure** **6**A). The spontaneous release for different gel concentrations (i.e., different mesh sizes) was also tested and found negligible between a concentration of 8% and 17.5%. (Figure 6B).

Next, we evaluated the hydrogels’ antibacterial efficacy by exposing their surfaces to bacterial suspension. 100 µL of gels at different concentrations (8%, 12.5%, and 17.5%), without and with loaded vancomycin, were prepared in the wells of a 96‐well plate and then challenged with bacteria (5 µL K‐12 *E. coli* culture, 100 µL LB, starting OD600 was 0.01). Hydrogels loaded with vancomycin showed an effective antibacterial activity against *E. coli* after 6 h. Hydrogels acting as a topical cover over an infected wound should also prevent the penetration of further bacteria and act as a block to any bacterial propagation. Hence, a hydrogel‐coated Transwell model with a pore size of 3 µm (large enough for *E. coli* to pass through) was placed on top of the wells of a 24‐well plate, and the bacterial solution was then placed on top of the hydrogels. We tested hydrogels with concentrations of 8% and 17.5%, both empty and loaded with vancomycin. We then grew CFUs from the media from the bottom of the well and calculated CFUs mL⁻^1^. We observed that bacterial penetration was blocked regardless of the component's concentration or the presence of vancomycin (Figure 6D). Finally, the long‐lasting effect of the hydrogel loaded with vancomycin was tested, compared to vancomycin and *E‐coli* as controls. The optical density at 600 nm was measured every 15 min for 20 h (Figure 6E). The vancomycin‐loaded hydrogels showed a long‐lasting antibacterial activity, and the hydrogel with the lowest concentration was the most effective. The empty hydrogels showed bacterial growth even above the *E. coli* control, with the lowest concentration hydrogel having the highest bacterial growth.

### 2.5 In Vivo Wound Healing and Toxicity Studies

**Figure 7:**
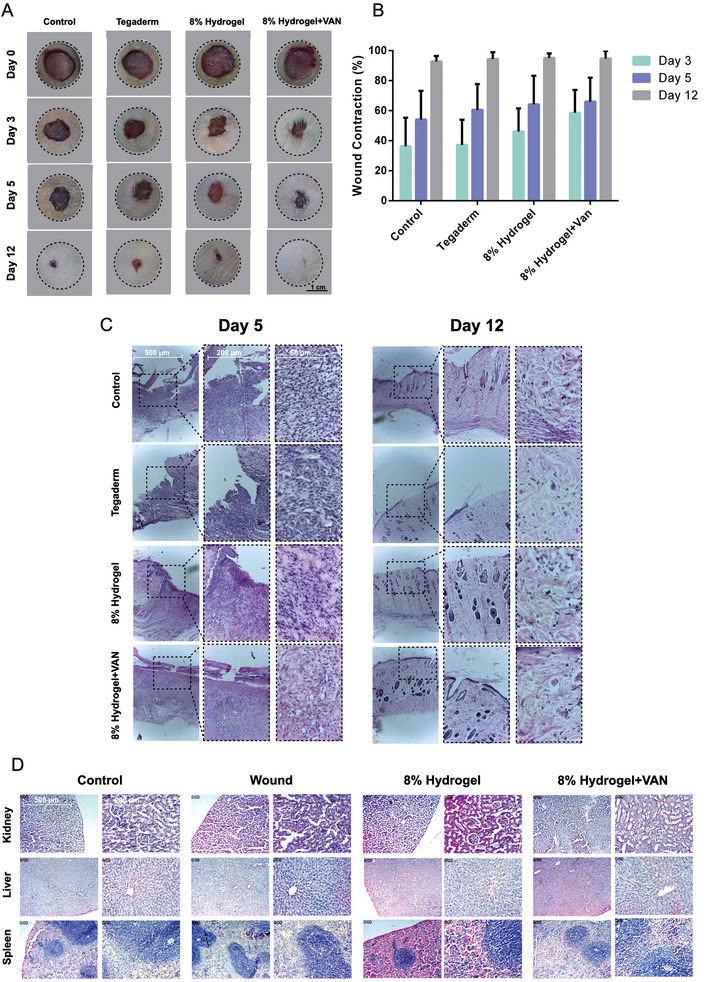
In‐vivo wound healing model: A) Photographs of wound cuts for control, Tegaderm, 8% hydrogel, and 8% hydrogel+VAN groups at day 0, 3, 5, and 12. B) Wound concentration in percentage determined by ImageJ for the groups after 3, 5, and 12 days. C) Hematoxylin‐eosin staining (H&E) of rat's wound tissues at days 5 and 12 for the same test groups. D) The histopathology results of H&E staining of the rat's main organs, including kidney, liver, and spleen.

As shown in the photographs of the wounds (**Figure** **7**A) and the percentage of wound contraction as determined by ImageJ software (Figure 7B), the Tegaderm and wound groups displayed slow healing during the 12‐days period. In fact, the results demonstrate that the healing progress speed was much higher in the 8% hydrogel and 8% hydrogel+VAN groups: 3 days after surgery, the percentage of wound contraction is 46.12±15% for 8% hydrogel and 58.65±15.1 for 8% hydrogel+VAN, respectively. By contrast, these values were 37.2±16.2% and 36.3 ±18% for the Tegaderm and control groups, respectively. Moreover, Figure 7C demonstrates the results of hematoxylin‐eosin staining (H&E) staining of rats’ wound tissues on day 5 and day 12 post‐surgery. The results on day 5 displayed many inflammatory cells and disorganized micro‐architectures in control groups compared to other groups. At day 12, the collagen fibers in 8% hydrogel and 8% hydrogel+VAN groups were more organized. In 8% hydrogel+VAN groups, a new layer of epidermis is observable. The histopathology results of H&E staining of the rat's main organs, including kidney, liver, and spleen (Figure 7D), did not show histopathological alterations such as dead tissue or alterations in the structure of cells. In addition, there were no signs of inflammation in 8% hydrogel, 8% hydrogel+VAN and Tegaderm groups compared to the control group. The hydrogels were degraded and absorbed by the wounds 7 days after administration.

Figure S11 (Supporting Information) demonstrates the results of blood hematological and biochemical factors. As shown in the graphs, biochemical factors, including total protein (TP), albumin (ALB), calcium (Ca), blood urea nitrogen (BUN), creatinine (CREA) are not significantly different in 8% Hydrogel and 8% Hydrogel+VAN. Moreover, white blood cells (WBCs), red blood cells (RBCs), hemoglobin (HGB), hematocrit (HCT) and platelets (PLT), mean corpuscular volume (MCV), mean corpuscular hemoglobin (MCH), and mean corpuscular hemoglobin concentration (MCHC) are not significantly different among different groups.

## 3 Conclusion

In this work, an antibacterial hydrogel was prepared on a multigram scale using a straightforward process. The hydrogel was loaded with an antibacterial peptide for topical delivery to infectious wound sites and to prevent bacterial contamination. Due to the incorporated disulfide bonds, the hydrogel backbone can be degraded in infectious wounds in response to reductive or oxidative environment, releasing its cargo of the antibacterial peptide; the hydrogel will eventually be removed after degradation, without causing any secondary damage to the wound. The in vitro results show that the hydrogel and its degradation products are non‐toxic to cells, and the hydrogel loaded with vancomycin acts as a barrier against bacteria, with a significant antibacterial effect against *E. coli*. The hydrogels loaded with vancomycin exhibited improved healing of infected wounds in an in vivo dermatological mouse model. The high biocompatibility, degradability, and antibacterial properties of the hydrogel qualify it as a promising wound dressing for topical drug delivery to infected wounds.

## 4 Experimental Section

### Materials

Glycidol (96.0%) from Sigma‐Aldrich (distilled) and 3,3′‐dithiodipropionic Acid (>99.0%) from Tokyo Chemical Industry (TCI) were used for the polymer synthesis. Acryloyl chloride (96.0% stabilized with phenothiazine) purchased from ABCR and Sodium hydride (60.0% dispersion in mineral oil in soluble bags), from ACROS Organics were used for the acrylation. Albumin‐fluorescein isothiocyanate conjugate and vancomycin hydrochloride purchased from Sigma‐Aldrich were encapsulated in the hydrogels. For the release studies, Glutathione (GSH, 98.0%) and DL‐1,4‐Dithiotreitol (DTT, 98.0%) from Thermo Scientific were the reducing agents employed. The PBS solution was prepared with 1x PBS tablets for 1000 mL from ChemSolute, Th. Geyer. Methanol (MeOH, ≥99.9%) and anhydrous dimethyl ether (DMF) were purchased from Acros Organics. Cellulose ester dialysis membrane (MWCO = 1.0 kDa) was purchased from Carl Roth GmbH + Co.

### Instrumentation

^1^H NMR and ^13^C NMR spectra were recorded either on a Bruker AVANCE III 500 (Bruker Corporation), or a Jeol ECP 600 (JEOL GmbH). Chemical shifts are reported in δ (ppm) and referenced to the respective solvent. Deuterated water (D_2_O) was used for the characterization of the final polymeric products. Infrared spectroscopy measurements of the compounds were conducted using a JASCO FT/IR‐6700 FT‐IR Spectrometer. For the encapsulation and release experiments, UV–vis measurements were conducted on an Agilent Cary 8454 UV–visible spectrophotometer, using semi‐micro reusable cuvettes. The rheological trends of the hydrogels were evaluated by using a Malvern Kinexus rheometer. A plate upper geometry (8 mm diameter) and a cone upper geometry (1:20 mm diameter) were used as test geometries depending on the viscosity of the hydrogels. Time and frequency sweep tests were conducted before the measurements and fixed at 1 Hz and 1% strain. A closed system was used to avoid evaporation of water from the hydrogels during measurements. The surface morphologies of lyophilized hydrogel samples were analyzed using a Scanning Electron Microscope (SEM), with an SU8030 Hitachi instrument, operating at an accelerating voltage of 15 kV.

### Synthesis of Reducible Hyperbranched Polyglycerol (hPG‐DTDPA)

To a 100‐mL 3‐neck Schlenk flask equipped with a mechanical stirrer, 4.62 g (0.022 mol) 3,3′‐dithiodipropanoic acid (DTDPA) was added, dried under high vacuum for 1 h and flushed with Argon. 5, 10, or 20 equiv. amounts of glycidol was then added and the reaction was left to stir at 100 rpm at 70 °C for 3 days. Afterward, the reaction was quenched with water/methanol (1:1 v/v) and dialyzed against the same solution for the first day, changing the medium three times per day and eventually against only water for 2 days using a membrane with a MWCO of 1 kDa. The purified product was concentrated under reduced pressure and lyophilized to obtain the final product.

For the Acrylation, 2.54 g of SS‐hPG was dried in a 50‐mL Schlenk flask under high vacuum at 40 °C overnight. The next day, the polymer was flushed with Argon and left to cool off before dissolving it in 10 mL of anhydrous DMF. 205 mg (0.15 eq / OH groups) Sodium hydride was then added to the flask at 0 °C. The reaction was left to stir for an hour to enable the deprotonation of the hydroxyl groups. Subsequently, 0.46 g (0.15 eq / OH groups) of acryloyl chloride was added dropwise and the reaction was left to stir at room temperature overnight. Afterward, the reaction was dialyzed against water for 2 days using a 1 kDa MWCO membrane. The final product was concentrated and lyophilized and then redissolved in phosphate buffered saline solution (PBS) for a 100  mg /100 µL concentration stock solution and stored at −4 °C. The degree of acrylation was calculated according to the ^1^H NMR spectrum and was found to be 6.5% (Equation S2, Supporting Information).

### Synthesis of 4‐Arm‐PEG‐Thiol

4‐arm‐PEG‐thiol was synthesized in accordance with what is previously published in the literature.^[^ ^20^ ^]^ 4‐arm‐PEG was functionalized with thiol group by using thiourea. First, a mesyl group was introduced, then the thiolation and hydrolysis were carried out at 80 °C. After purification, a pale yellowish precipitate was obtained in a high yield. The final product was characterized by ^1^H NMR and the functionalized thiol groups were quantified by Ellman's assay, which showed ≈3.7 groups per molecule (Page S4‐S6).

### Hydrogel Chemical Crosslinking

Hydrogels were prepared for different building block and crosslinker concentrations (8%, 10%, 12.5%, and 17.5%) and ratios (3:1, 2:1, 1:1, 1:2, and 3:1) to define their viscoelastic properties through rheology measurements. The hydrogel is formed via the thiol‐ene click reaction between the acrylate groups of the hPG and the thiol groups of the PEG in PBS solution at a pH of 7.4. The general procedure consisted of preparing two solutions of SS‐hPG‐Acry and 4‐arm‐PEG‐thiol in PBS solution. 100 µL of 4‐arm‐PEG‐thiol was first added to a 2‐mL Eppendorf cap, followed by 100 µL SS‐hPG‐Acry solution and mixed with the help of an Eppendorf pipette. The cap was then sealed with the inversed Eppendorf and the hydrogels were left to shake at 100 rpm overnight.

### Hydrogel Swelling

Freshly prepared hydrogel samples (200 mg) were freeze‐dried and immersed in deionized water (5 mL) at room temperature. At periodic time intervals, the weight of the hydrogels was recorded after removing the surface water. The swelling percentage was calculated as the percentage of weight increase of the swollen state at an interval t (*W*_t_‐*W*_0_) with respect to the initial weight of the dry hydrogel (*W*_0_) (Equation 1). Swelling experiments were repeated for three different samples (n = 3) and plotted as Mean ± SD.

(1) $$\%swelling\;=\frac{W_{t}-W_{0}}{W_{0}}\;\times 100$$

### Hydrogel Self‐Healing Test

The 8% and 17.5% (w/v) hydrogels (with and without a blue food dye) were prepared in the Eppendorf cap as mentioned previously. After their formation, the gels were cut in half using a blade. One of each half was then pressed into each other in the same orientation as the cut. The combined hydrogels were observed at t = 0, 3, 6, and 24 h to visualize the self‐healing properties.

For rheological assessment of self‐healing, the 17.5% hydrogel subjected to high (600%) and low (1%) strains in cycles for a total of three cycles. G′ and G″were recorded and plotted with respect to time to assess the conservation of hydrogel properties throughout the cycles.

### Hydrogel Mesh Size Calculation

The mesh size of the hydrogels with different concentrations was calculated using the Canal‐Peppas equation based on the elastic modulus of the gel ^[^ ^21^ ^]^ according to Equation 2.

(2) $$\xi \;={\left(\frac{RT}{G^{\prime }N_{A}}\right)}^{\frac{1}{3}}\;$$

where R is the gas constant in J mol^−1^K^−1^, T is the temperature in K, G′ is the elastic modulus N m^−2^, and N_A_ is Avogadro's constant.

### Hydrogel Redox‐Responsive Degradation

Rheological experiments were carried out to demonstrate the disassociation of the gel matrix in the presence of a reducing agent. 17.5% hydrogels were incubated in 1 mL of PBS, 10 mM GSH, and 10 mM DTT media for different time points. At each time point the degradation of the gel was investigated using time sweep tests on a rheometer recording G′ and G″. Simultaneously, 0.5 mL of each media at a certain time point was removed and lyophilized, and the degradation was assessed based on the weight loss of the initial hydrogel. For visual assessment of degradation, two fluorescein isothiocyanate‐labeled bovine serum albumin (FITC‐BSA) loaded hydrogels were incubated in PBS and 10 mM DTT solutions respectively. Photographs of vials were taken under UV illumination (365 nm) to indicate the gel degradation.

### Active Pharmaceutical Ingredient Loading and Release Studies

FITC‐BSA and vancomycin were used as a model protein and antimicrobial glycopeptide as active pharmaceutical ingredient (API) respectively. The APIs were encapsulated into hydrogels during gelation. In short, a concentration of 1 mg 50 µL of API, 17.5 mg/50 µL of 4‐arm‐PEG‐thiol and 17.5/100 µL of hPG‐DTDPA were prepared in PBS solution. In an Eppendorf's cap the 4‐arm‐PEG, API and hPG‐DTDPA were added sequentially. The gel was mixed, sealed, and left to shake overnight. After gelation, hydrogels were placed into 5 mL of PBS, 10 mM GSH, and 10 mM DTT containing vials. At different time points, 2 mL samples were taken out and the amount of API released in the supernatant was determined using a UV−vis spectrophotometer at 495 nm and 280 nm for FITC‐BSA and vancomycin respectively. For the on‐demand release study, hydrogels were treated with 100 mM GSH and 100 mM DTT after 3 h of passive release. The release experiment was repeated three times for each set of hydrogels.

### In Vitro Cytotoxicity Assays

Cytotoxicity of the hydrogels with different concentrations with and without addition of GSH was investigated via a PrestoBlue viability assay on L929 mouse fibroblast cells. Cells (69000 cells/well) were seeded on a 12‐well plate with 2 mL of the culture medium and incubated at 37 °C overnight to grow and adhere. Transwell with a pore size of 0.45 µm was inserted on top of the plate's well. 200 mg hydrogels (17.5%, 12.5%, and 8%) with and without the addition of 5 mM GSH were placed on inside the Transwell. The hydrogels were incubated in the cells for 24 and 96 h. After the incubation time, 100 µL was taken of each well in three replicates and treated with 10% of PrestoBlue solution for 3 h, and the absorbance values at 570 nm were measured using a microplate reader. Non‐treated cells were used as a positive control, and cells treated with 5 mM GSH were used as a control for the degradation experiment.

### Live/Dead Cell Viability Assay

The L929 mouse fibroblast cells adhered on the previous 12‐well plate were rinsed twice with PBS and the cells were stained according to the protocol of the live/dead assay kit (Sigma, 04511‐1KT‐F). The cells were stained with PBS solution containing 10 µL of calcein‐AM and then with 5 µL of propidium iodide (PI) for 30 min at 37 °C. After removing the solution and washing with PBS, the cells were imaged using a fluorescence microscope (Zeiss Observer A1 equipped with AxioCam MRc5) and AxioVision software. The live and dead cells exhibited green and red fluorescence respectively due to calcein‐AM and PI.

### In Vitro Antibacterial Activity and Growth Curves

In this study the K‐12 *E. coli* DH5α was streaked out on LB agar from frozen stock. Liquid culture was created from a single colony isolated from the LB plates and allowed to grow for 6 h at 37 °C in 5 mL of LB media. 100 µL of LB media was added on top of 100 µL of solidified hydrogel of varying concentrations with the presence or absence of vancomycin in a 96‐well plate (round bottom, Sarstedt), followed by 5 µL of bacterial culture. The lid of the 96‐well plate was coated in anti‐fog solution (0.05% TritonX‐100, 20% Ethanol in water) ^[^ ^22^ ^]^ and allowed to completely dry under the clean bench hood. The closed with lid 96‐well plate was placed in the Agilent BioTek Epoch 2 Microplate Spectrophotometer and allowed to grow for 20 h at 37 °C with continuous shaking (double orbital) measuring at OD_600_ in 15 min intervals. The growth curves experiments consisted of three biological repeats with a further subset of three technical repeats for each of the biological repeats (N = 9).

Colony forming units (CFUs) were then preformed after the 20‐h growth curve using the resultant culture as described in Maan et al.^[^ ^23^ ^]^ Briefly, the culture was transferred to a 96‐well plate and a serial dilution from 10^0^ to 10^−7^ was performed in DPBS. 20 µL of varying dilutions were spotted onto LB agar plates and allowed to dry completely in clean bench hood. Plates were incubated overnight at 37 °C and CFUs were then counted. The CFU experiments consisted of five biological repeats with a further subset of three technical repeats for each of the biological repeats (N = 15).

### Bacterial‐Blockage Activity

Hydrogels of concentrations 17.5% and 8% with the presence and absence of vancomycin were formed in parafilm wrapped Transwell (pore size 3 µm) and hardened overnight. Parafilm was carefully removed from Transwell in clean bench hood and Transwell were placed in 24‐well plate that contained 100 µL of L B media (The Transwell was partially bathing in the 100 µL of the LB). 100 µL of LB media was then added to the top of each Transwell followed by 5 µL of *E. coli* culture (prepared in the same way as described above). Plates were covered and incubated overnight at 37 °C in static conditions. The following day, Transwells were carefully removed and culture from 100 µL of LB was transferred to a 96‐well plate and CFUs were performed as described above. These experiments consisted of four biological repeats with a further subset of three technical repeats for each of the biological repeats (N = 12).

### In Vivo Wound Healing and Toxicity Studies

In all in vivo studies, the rats were kept in their houses for one week to acclimate them to the new conditions. During the studies, the cycle of 12 h of light and 12 h of darkness was maintained in the house, as well as the ambient temperature of 21–23 °C and relative humidity of 50–60%. All principles of working with laboratory rats and methods were in accordance with the ethical standards and instructions of the Committee for Working with Laboratory Animals of Lorestan University of Medical Sciences (Ethical code: IR.LUMS.REC.1402.213). *S. aureus* strain (ATCC 25 923)‐infected full thickness skin was employed to investigate the wound healing potential of hydrogels in a rat model. For this purpose, rats (male Sprague‐Dawley rats, 180–220 g) were randomly divided into four groups (*n* = 6) including wound alone without any treatment (Wound), the commercially‐available dressing as a positive control (Tegaderm), Hydrogel (8% Hydrogel), and drug‐loaded hydrogel (8% Hydrogel+VAN). Before the wound creation, the rats were anesthetized with a mixture of ketamine and xylazine. Then, two wounds were created on the back side of animals with a diameter of 10 mm after shaving them. Afterward, 20 µL of a0.5 MaFarland *S. aureus* suspensions were employed for each wound to infect it. After 15 min, the related treatments were employed in each group. The wounds in each group were monitored and photographed on days 0, 3, 5, and 12. The images were analyzed by ImageJ software. on the 5 and 12th days, the wound and the surrounding repaired tissue were harvested and fixed in 4% paraformaldehyde. After dehydration with respectively: alcohol 70%, 80%, 90%, absolute 1 and absolute 2, the samples were embedded in paraffin, section with 5–7 µm thickness were obtained by a microtome, finally, the samples were stained by H&E to investigate the wound repair and inflammation.

To investigate the animal toxicity of hydrogel, in addition to measuring the toxic effect of hydrogel and drug‐containing hydrogel on important organs such as kidney, liver, and spleen, the function of these organs is also evaluated. For this purpose, on day 12, 2 mL of blood was taken to check hematological parameters including White blood cells (WBCs), Red blood cells (RBCs), hemoglobin (HGB), Hematocrit (HCT) and Platelets (PLT), Mean corpuscular volume (MCV), mean corpuscular hemoglobin (MCH), mean corpuscular hemoglobin concentration (MCHC), and biochemical factors including total protein (TP), albumin (ALB), calcium (Ca), phosphorus (ph), blood urea nitrogen (BUN), creatinine (CREA). Moreover, to evaluate the morphological changes in the main organs of animals including kidney, liver, and spleen, the tissues of these organs were harvested and first fixed in 4% paraformaldehyde, dehydrated, embedded in melted paraffin, cut into thin slices with a microtome, and stained with H&E and was observed under an optical microscope.

### Statistical Analysis

The experiments were performed with at least three biological repeats with three technical repeats within each biological repeat. Error bars represent ±SD. Statistical analysis was executed using the GraphPad Prism software (GraphPad Software, Inc., San Diego, CA). Data analysis was performed using SPSS Statistics version 24. The data were analyzed using the and one‐ way ANOVA followed by Tukey's test. Levels of statistical significance were displayed with *p < 0.05.

## Conflict of Interest

The authors declare no conflict of interest.

## Supporting information

Supporting Information

## Data Availability

The data that support the findings of this study are available in the Supporting Information of this article.
