# Supplementary material for: Redox‐Responsive Hydrogels Loaded with an Antibacterial Peptide as Controlled Drug Delivery for Healing Infectious Wounds
Source: Adv Healthc Mater. 2024 Jul 8;13(27):2401289. doi: 10.1002/adhm.202401289 (PMC12344618; doi:10.1002/adhm.202401289)
Supplement: Supplementary file 1 — Supporting Information [file ADHM-13-0-s001.docx]

Supporting Information

**Redox-Responsive Hydrogels Loaded with an Antibacterial Peptide as Controlled Drug Delivery for Infectious Wound Healing**

*Mariam Cherri, Paraskevi S. Stergiou, Zainab Ahmadian, Tatyana L. Povolotsky, Boonya Thongrom, Xin Fan, Ehsan Mohammadifar*, Rainer Haag**

**
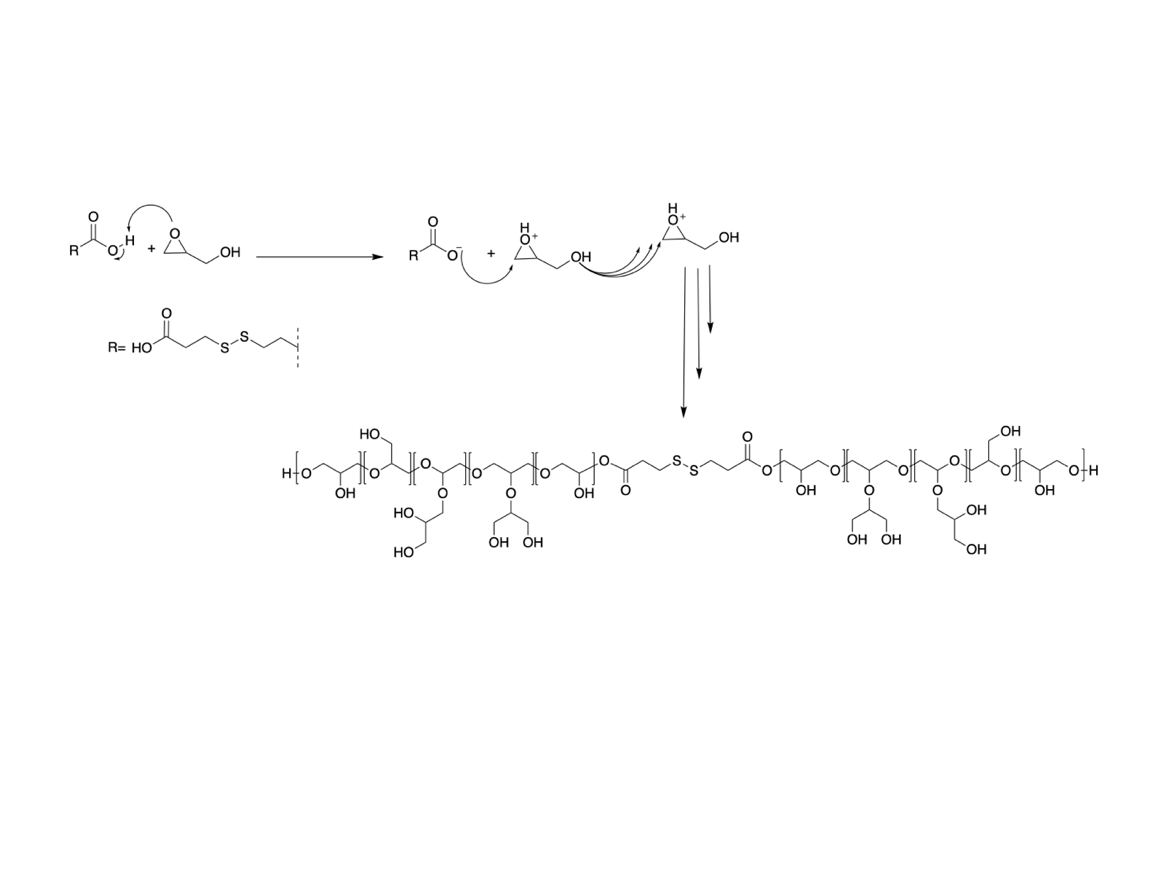
**

**Scheme S1.** Suggested reaction mechanism of the activated monomer (AM) cationic ring opening polymerization of glycidol with DTDPA acting as an initiator.

**Synthesis and characterization of hPG-DTDPA**


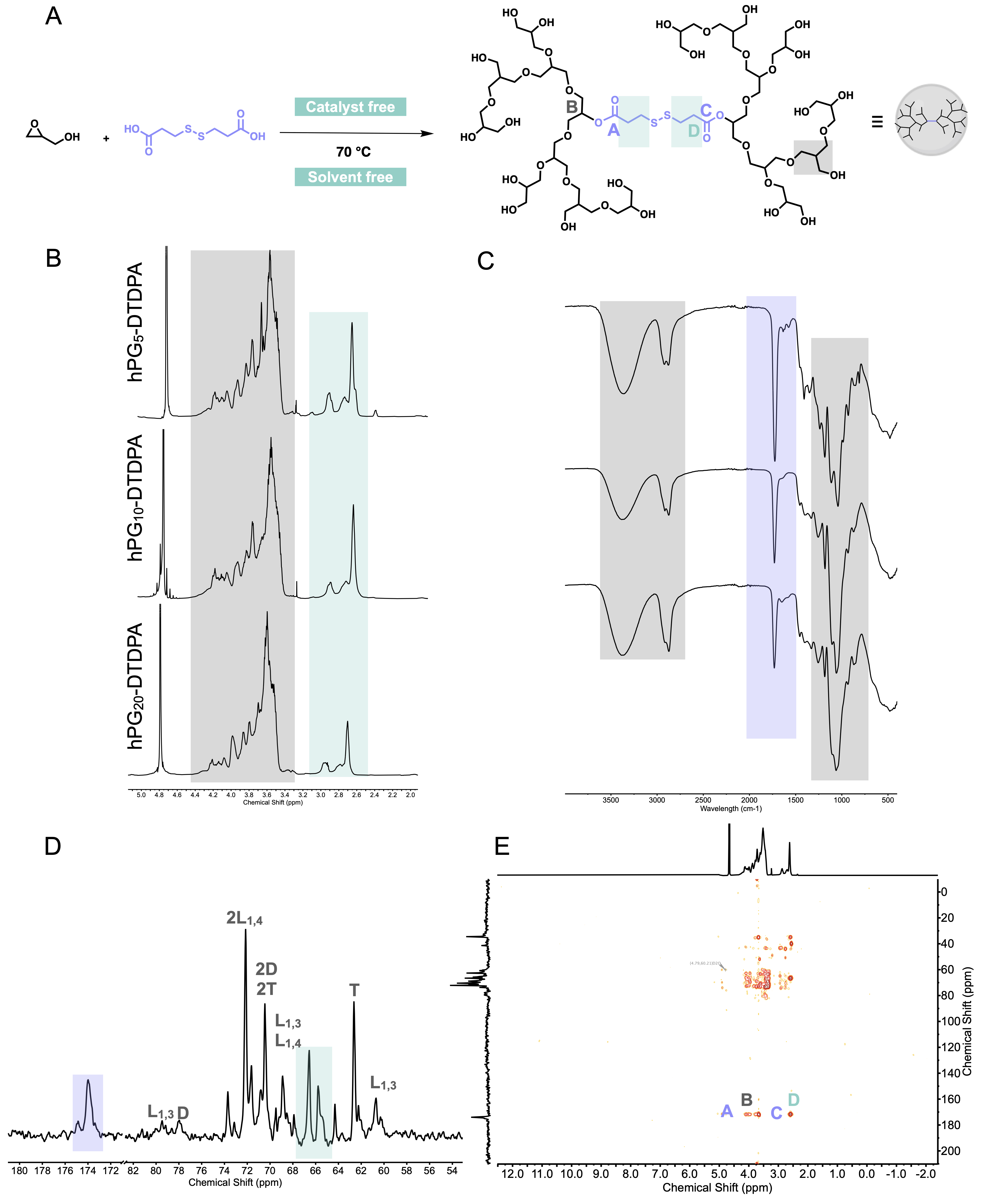


**Figure S1.** (A) Synthesis’s scheme of the solvent-free, catalyst-free activated monomer cationic ring opening polymerization of glycidol using DTDPA, (B) and (C) ^1^H NMR and FTIR spectra of hPG-DTDPA for different molar ratios [Gly]:[DTDPA] respectively, and (D) and (E) inverse-gated ^13^C NMR and HMBC spectra of hPG_10_-DTDPA in D_2_O in 500 or 600 Hz magnetic field.

Table S1. Characterization of reducible hPGs synthesized by different feed molar ratios of [Gly]:[DTDPA]

|  | hPG_5_-DTDPA | hPG_10_-DTDPA | hPG_20_-DTDPA |
| --- | --- | --- | --- |
| Molar ratio of [Gly]:[DTDPA] | 5:1 | 10:1 | 20:1 |
| Degree of branching^a)^ | 0.33 | 0.35 | 0.18 |
| Terminal units^a)^ (%) | 27 | 43 | 61 |
| Dendritic units^a)^ (%) | 14 | 12 | 4 |
| Linear 1,3 units^a)^ (%) | 11 | 7 | 6 |
| Linear 1,4 units^a)^ (%) | 47 | 43 | 61 |
| M_n_^b)^ (g/mol) | 1600 | 2000 | 2400 |
| Disulfide content^b)^ (%) | 12.5 | 10.0 | 4.76 |
| Disulfide content^c)^ (%) | 14.16 | 11.08 | 4.76 |
| Yield (%) | 30 | 62 | 71 |

^a)^Inverse-gated ^13^C NMR in D_2_O, ^b)1^H NMR in D_2_O and ^c)^elemental analysis.

**Figure S2.** FTIR spectra of DTDPA showing the carbonyl stretch of the carboxylic acid at 1630 cm^-1^.

**^1^H NMR molecular weight calculation**

The molecular weight of the polymer is calculated by fixing the protons corresponding to DTDPA to 8.00, since there is only one molecule of DTDPA incorporated in the polymer. The protons belonging to the polyglycidol backbone (3.4 – 4.4 ppm) were divided by 5 (number of protons in a repeating unit of glycidol) to calculate the number of glycidol repeating unit. The number of glycidol repeating units was then multiplied by the molecular weight of one repeating unit (74.08 g/mol) and added to the molecular weight of DTDPA (395.35 g/mol) to have the total molecular weight of the polymer (Equation S1).

| $M_{n}(g/{mol})=\frac{\int3.4-4.4 ppm}{5}\times M_{gly}+M_{DTDPA}$ | S1 |
| --- | --- |

**Degradation of SS-hPG with TCEP**

**
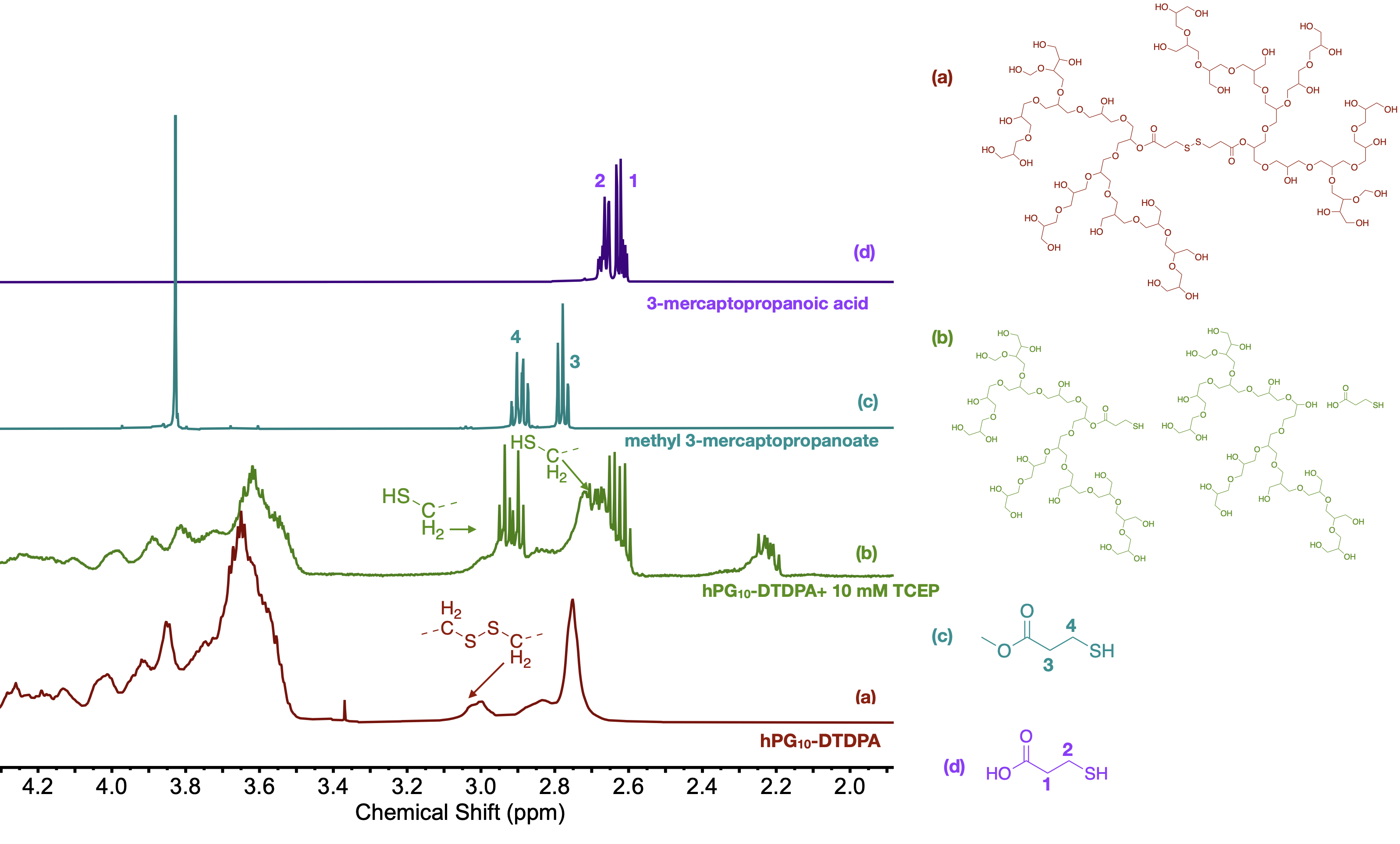
**

**Figure S3.** ^1^H NMR in D_2_O and structure corresponding to hPG_10_-DTDPA (a), hPG_10_-DTDPA + 10 mM TCEP (b), methyl 3-mercaptopropanoate (c) and 3-mercaptopropanoic acid (d). The objective was to identify the degradation products of hPG_10_-DTDPA after its incubation with 10 mM TCEP.

To identify the correspondent of this peak, methyl 3-mercaptopropanoate and 3-mercaptopropionic acid ^1^H NMR were recorded in D_2_O. The reason is the presence of a methylene group adjacent to a thiol in both molecules and a methylene group adjacent to an ester and to a carboxylic acid in methyl 3-mercaptopropanoate and 3-mercaptopropionic respectively. Observing the spectra, the peaks of the protons of the methylene group adjacent to the thiol in both molecules overlap with the peaks in the degradation spectra. In addition, the peaks of the protons of the methylene groups adjacent to an ester and carboxylic acid overlap as well. An acidic medium will partially hydrolyze the ester bonds, and knowing that TCEP will make the medium acidic, we except it to also hydrolyze the ester bonds partially in addition to cleaving the disulfide bond. Having the peaks overlap between the degradation spectra and the methyl 3-mercaptopropaoate and the 3-mercaptopropanoic acid spectra, can indicate that the presence of thiols, esters, and carboxylic acids in the degradation products.

**Degree of acrylation calculation (dAcrylate %)**

The degree of acrylation was calculated by fixing the integral of the protons corresponding to the glycidol (3.4-4.4 ppm) to 5.0 (number of protons in one glycidol repeating unit) to obtain the integral of the protons between 6.0 and 6.6. these protons belong to the alkene of the acrylate. This integral was then divided by 3 (number of protons in one alkene unit).

| $dAcrylate (\%)=\frac{\int6.0-6.6 ppm}{3}\times100$ | S2 |
| --- | --- |

**4-arm-PEG-thiol *synthesis & characterization***

**Scheme S2.** Synthesis scheme of 4-arm-PEG-thiol.

*Materials and Methods:* Chemicals and solvents were purchased from Merck KGaA, Darmstadt, Germany and used directly without any purification unless stated otherwise. Diethyl ether (100%) was purchased from VWR chemicals. Potassium hydroxide (99.5%), and dichloromethane (DCM, 99%) were purchased from Fischer Scientific. 4-arm PEG 10 kDa was purchased from JenKem Technology USA Inc. Tris(2-carboxyethyl) phosphine hydrochloride (TCEP) was purchased from TCI Deutschland GmbH. All NMR spectra (1H and 13C) were recorded at 300 K by a Jeol Eclipse 500 MHz (Tokyo, Japan) or a Bruker AVANCE III 700 MHz spectrometer (Billerica, MA, USA). Chemical shifts δ were reported in ppm and the deuterated solvent peak was used as a standard. The determination of thiol group was performed by an Agilent Cary 8454 UV/visible spectrophotometer using disposable semi-micro-UV cuvette.

*Synthesis of 4-arm PEG mesylate:* 10 kDa dried 4-arm PEG OH (Figure S1, 7 g, 0.7 mmol, 1 eq.) was dissolved in anhydrous dichloromethane (DCM, 50 mL) and TEA (0.97 mL, 7 mmol, 10 eq.) was added to the reaction flask. The mixture was cooled in an ice bath and methane sulfonyl chloride (0.43 mL, 5.6 mmol, 8 eq.) was added dropwise. The reaction was left to stir for 24 h. Afterwards, the crude product was washed three time with brine, dried with Na_2_SO_4,_ and concentrated under high vacuum. The crude mixture was precipitated in cooled diethyl ether, collected, and dried overnight under high vacuum. The precipitate product was obtained as a white powder with an 85% isolated yield (Figure S2).


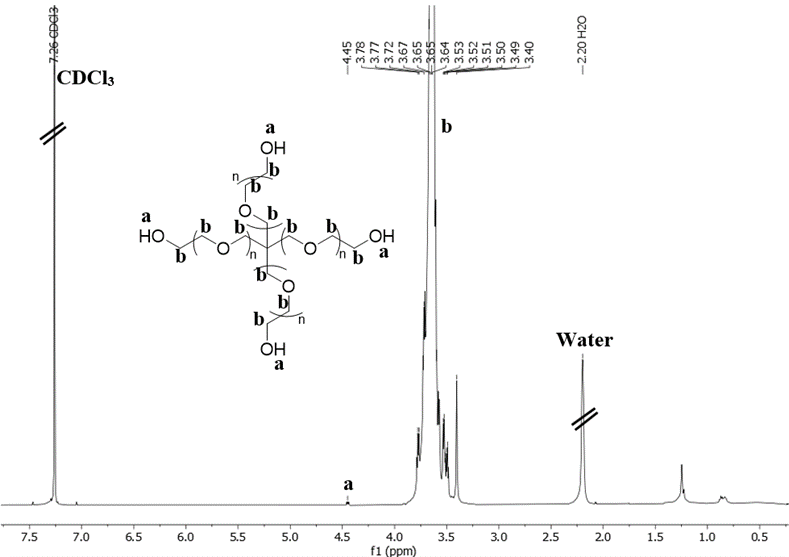


**Figure S4.** ^1^H NMR of 4-arm PEG in CDCl_3_.


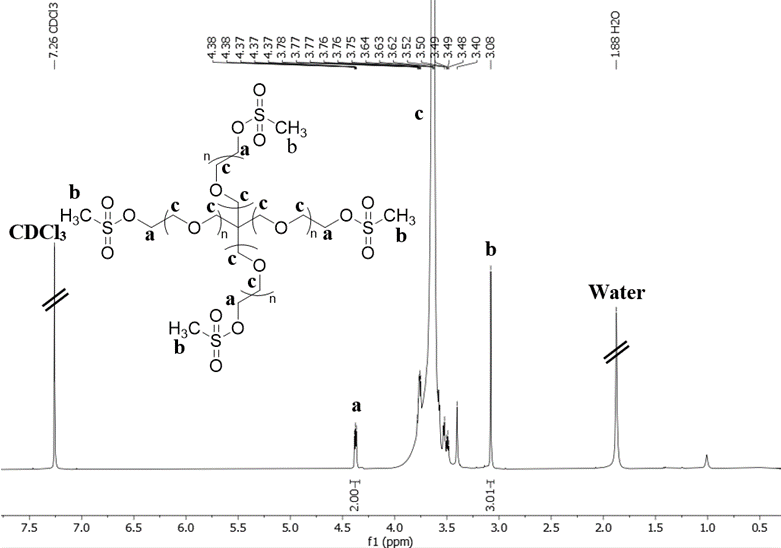


**Figure S5.** ^1^H NMR of 4-arm PEG mesylate in CDCl_3_.

*Synthesis of 4-arm PEG thiol*: 4-arm PEG mesylate (4.33 g, 0.43 mmol, 1 eq.) and thiourea (0.66 g, 8.7 mmol, 20 eq.) were added to the reaction flask containing 1-propanol (10 mL). The reaction stirred for 24 h at 80 °C to obtain the 4-arm PEG isothiouronium intermediate. After removing 1-propanol, KOH (0.024 g, 0.43 mmol, 4 eq.) and water (40 mL) were added to the reaction flask and the solution was heated again to 80 °C for 24 h. TCEP (0.5 g, 1.7 mmol, 4 eq.) was added to the crude mixture that was stirred for 2 h. The product was purified by first saturating the crude mixture with NaCl, second extracting the product with DCM three times, and drying it with Na_2_SO_4_, third concentrating the DCM layer, and finally precipitating it in cooled diethyl ether. Dried 4-arm PEG thiol was obtained as a pale yellowish powder with a 90% isolated yield (Figure 3). Free thiol content was also characterized by Ellman’s assay following the protocol of Thermofischer Scientific. The number of thiol groups was quantified using the standard calibration curve of cysteine which contains 1 thiol group. The result shows that the number of thiol group on 4-arm PEG thiol is approximately 3.7 groups).


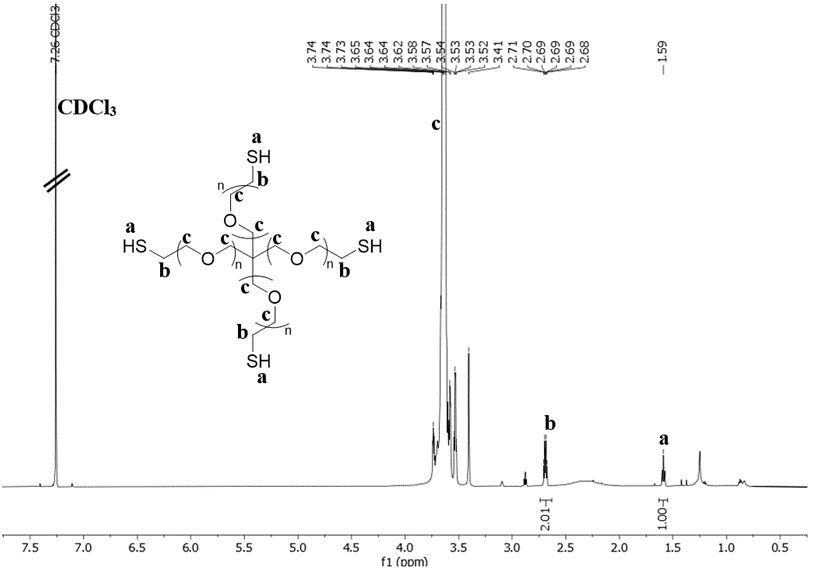


**Figure S6.** ^1^H NMR of 4-arm PEG thiol in CDCl_3_.

**Formation of the hydrogel**


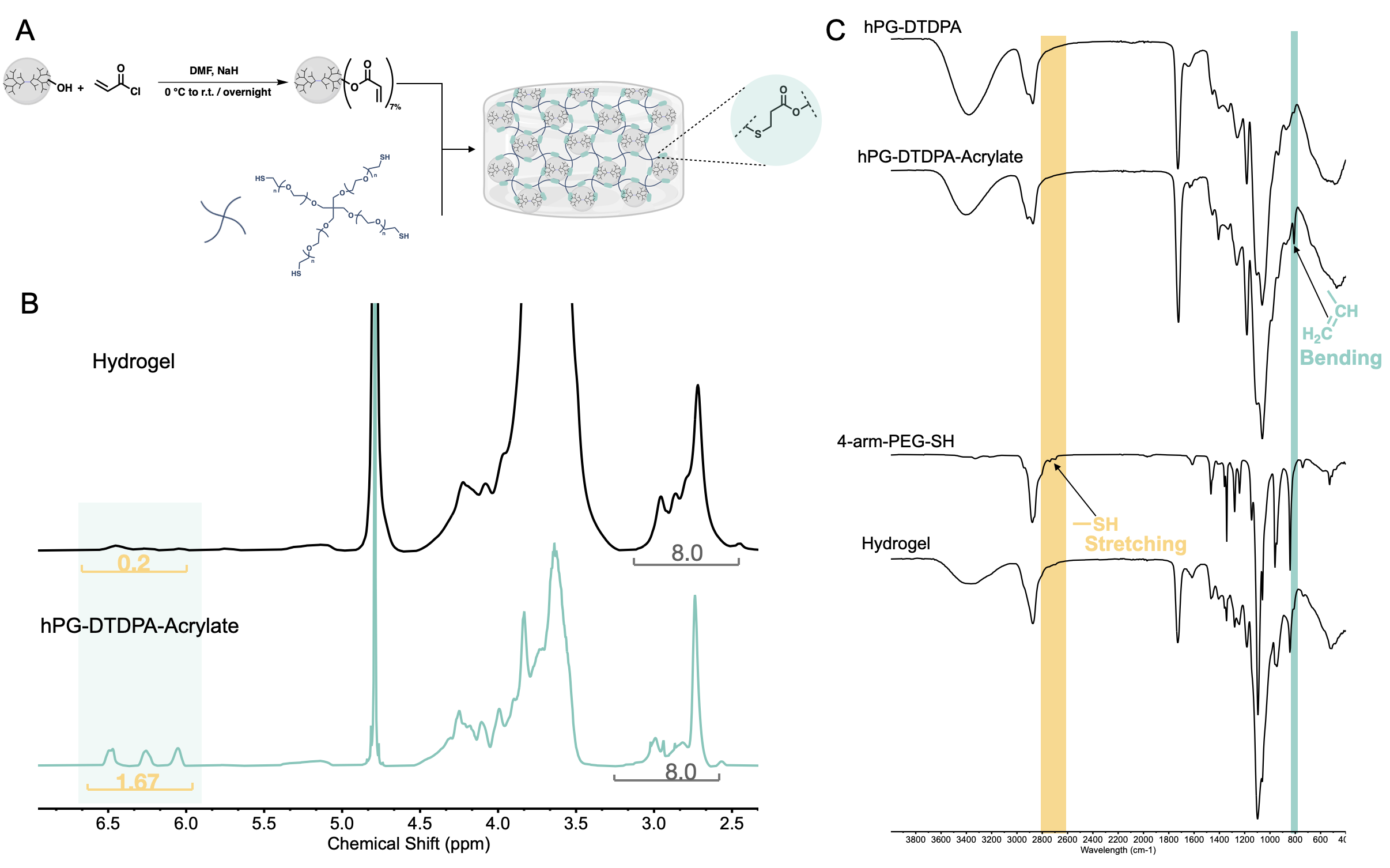


**Figure S7.** (A) Hydrogel’s synthesis scheme by first preforming acrylation of 7% of the hydroxyl groups of hPG_10_-DTDPA and crosslinking it with 4-arm-PEG-SH via a thiol-ene click reaction in PBS solution, (B) ^1^H NMR of hPG_10_-DTDPA-Acrylate and the formed hydrogel in deuterated PBS buffer, the disappearance of peaks corresponding to the alkene’s protons prove the occurrence of the click reaction in deuterated PBS buffer and (C) FTIR spectra of hPG_10_-DTDPA, hPG_10_-DTDPA-acrylate, 4-arm-PEG-SH and the formed hydrogel, the peaks corresponding the alkene bending and thiol stretching disappears in the formed hydrogel, proving the reaction.


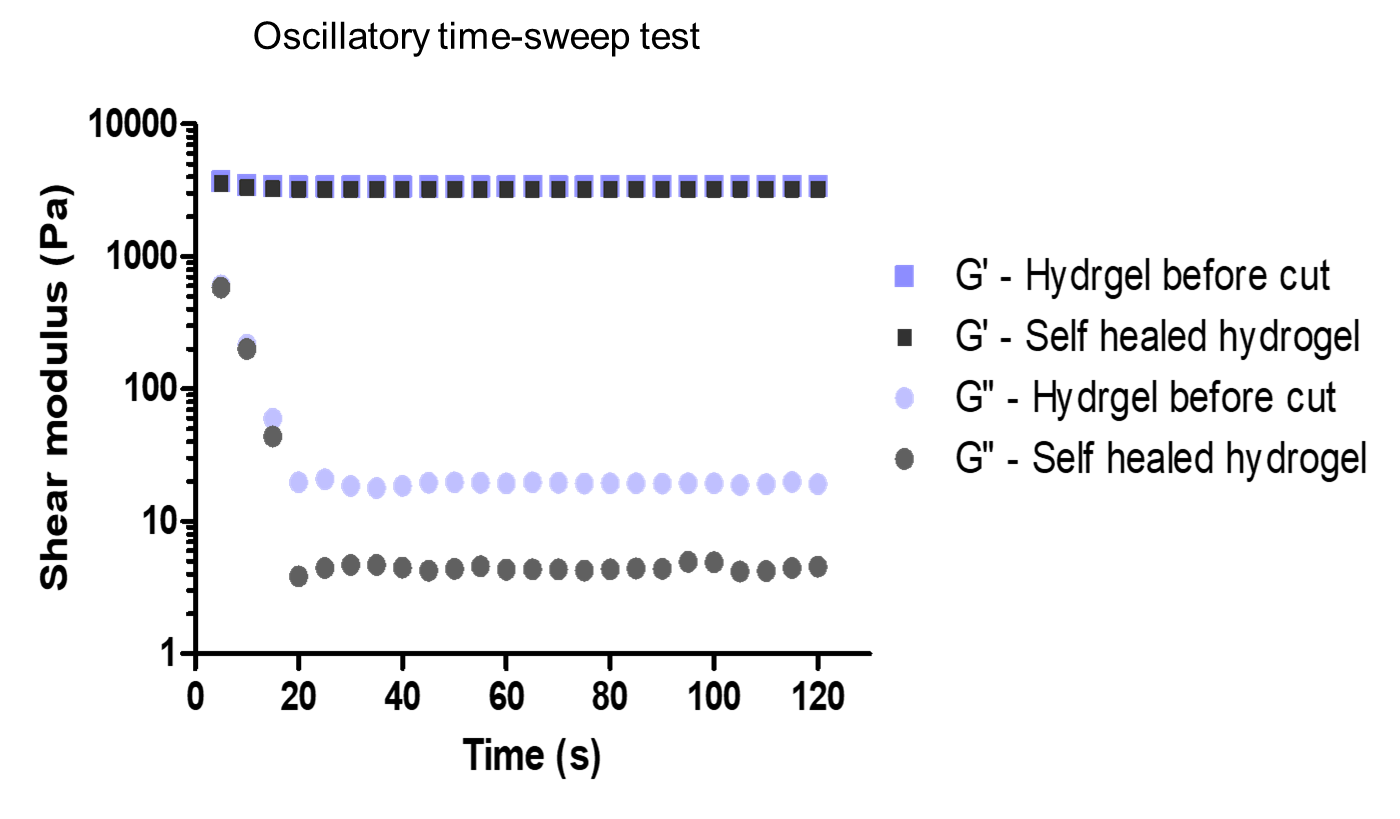


**Figure S8.** The rheological properties of hydrogels before and after self-healing.


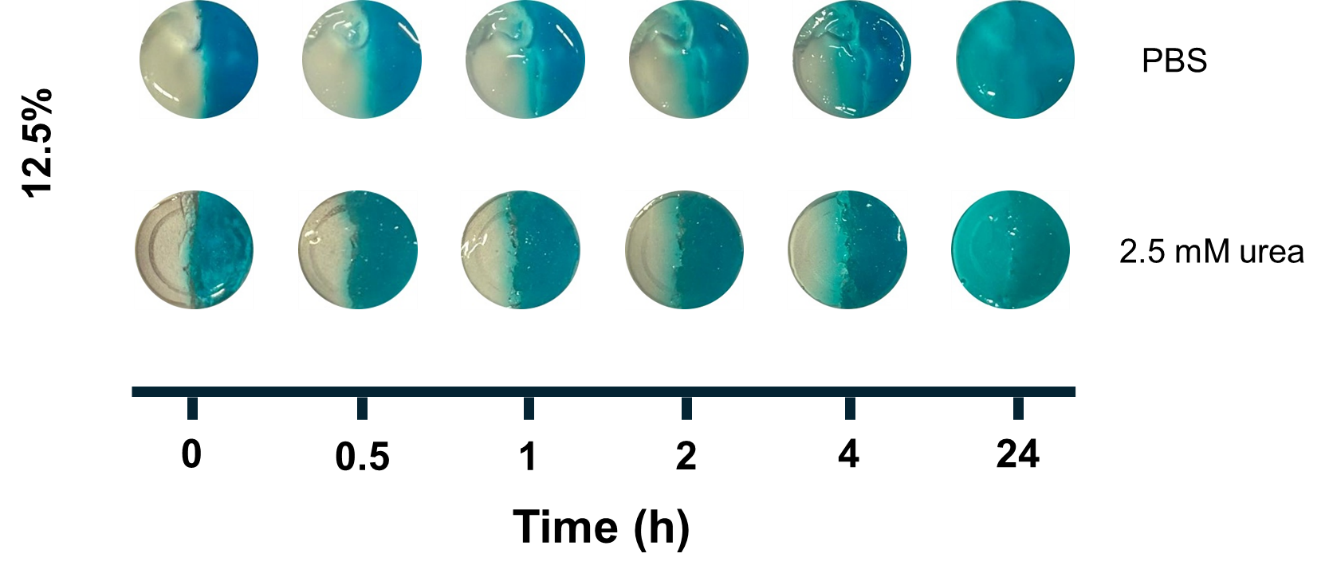


**Figure S9.** Photographs taken at different time points of 12.5% hydrogels in PBS and urea solutions, one half without dye and second half with blue dye, combined together to investigate the self-healing properties of the hydrogel.


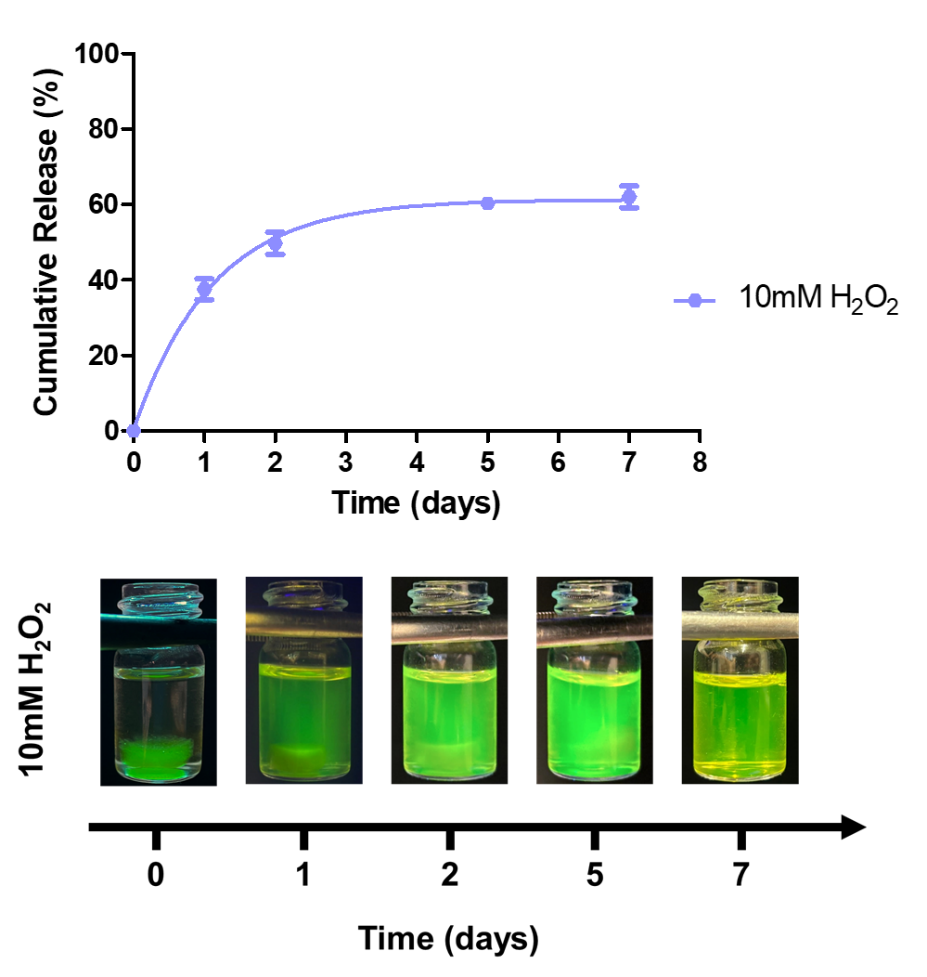


**Figure S10.** Release profile of FITC-BSA from 17.5% hydrogel in oxidative solution (10 mM H_2_O_2_ in PBS) (top), photographs of FITC-BSA-loaded 17.5% hydrogel incubated in 10 mM H_2_O_2_, captured at different time points (bottom).


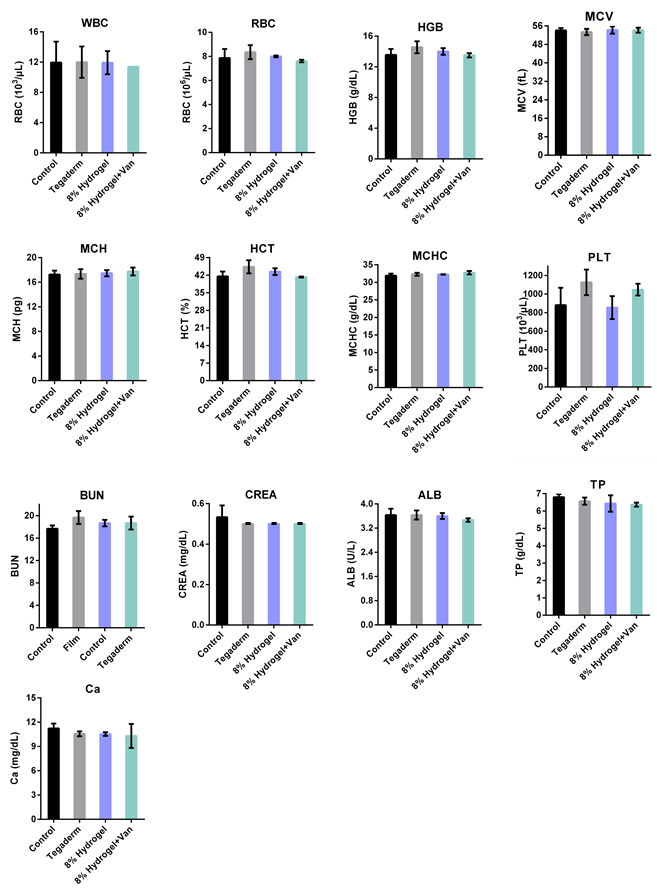


**Figure S11.** Blood hematological and biochemical factors for the control, Tegaderm, 8% Hydrogel and 8% Hydrogel+Van groups.
